# Antibacterial and COX-2 Inhibitory Tetrahydrobisbenzylisoquinoline Alkaloids from the Philippine Medicinal Plant *Phaeanthus ophthalmicus*

**DOI:** 10.3390/plants10030462

**Published:** 2021-03-01

**Authors:** Hilbert D. Magpantay, Ivane N. Malaluan, Joe Anthony H. Manzano, Mark Tristan Quimque, Kirstin Rhys Pueblos, Natalija Moor, Simon Budde, Porferio S. Bangcaya, Demi Lim-Valle, Hans-Martin Dahse, Abbas Khan, Dong-Qing Wei, Grecebio Jonathan D. Alejandro, Allan Patrick G. Macabeo

**Affiliations:** 1 Chemistry Department, De La Salle University, 2401 Taft Avenue, Manila 0922, Philippines; hilbert.magpantay@dlsu.edu.ph; 2 Laboratory for Organic Reactivity, Discovery and Synthesis (LORDS), Research Center for the Natural and Applied Sciences, University of Santo Tomas, España Blvd., Manila 1015, Philippines; inmalaluan@bicol-u.edu.ph (I.N.M.); joeanthony.manzano.sci@ust.edu.ph (J.A.H.M.); marktristan.quimque.gs@ust.edu.ph (M.T.Q.); kirstinrhys.pueblos.gs@ust.edu.ph (K.R.P.); 3 Chemistry Department, College of Science, Bicol University, Rizal St., Legazpi City 4500, Philippines; 4 Department of Biological Sciences, College of Science, University of Santo Tomas, España Blvd., Manila 1015, Philippines; 5 Chemistry Department, College of Science, MSU-Iligan State University, Iligan City 9200, Philippines; 6 Institut für Organische Chemie, Universität Regensburg, Universitätstrasse 31, D-93053 Regensburg, Germany; natalija.moor@chemie.uni-regensburg.de (N.M.); simon.budde@chemie.uni-regensburg.de (S.B.); 7 Biological Science Department, College of Teacher Education—University of Antique, Tario-Lim Memorial Campus, Tibiao, Antique 5707, Philippines; psbangcaya.ua@gmail.com; 8 Clinical Microbiology Laboratory, Department of Pathology and Laboratories, Makati Medical Center, Amorsolo St., Legaspi Village, Makati City 1229, Philippines; demivalle@yahoo.com; 9 Leibniz-Institute for Natural Product Research and Infection Biology, Hans-Knöll-Institute (HKI), D-07745 Jena, Germany; hans-martin.dahse@hki-jena.de; 10 Department of Bioinformatics and Biostatistics, State Key Laboratory of Microbial Metabolism, Shanghai Jiao Tong University, Shanghai 200240, China; abbaskhan@sjtu.edu.cn (A.K.); dqwei@sjtu.edu.cn (D.-Q.W.); 11 State Key Laboratory of Microbial Metabolism, Shanghai-Islamabad-Belgrade Joint Innovation Center on Antibacterial Resistances, Joint Laboratory of International Cooperation in Metabolic and Developmental Sciences, Ministry of Education and School of Life Sciences and Biotechnology, Shanghai Jiao Tong University, Shanghai 200240, China; 12 Peng Cheng Laboratory, Vanke Cloud City Phase I Bldg. 8, Xili St., Nashan District, Shenzhen 518055, China; 13 Plant Sciences Laboratory, Research Center for the Natural and Applied Sciences, University of Santo Tomas, España Blvd., Manila 1015, Philippines; gdalejandro@ust.edu.ph

**Keywords:** medicinal plants, ethnomedicinal, *Phaeanthus ophthalmicus*, limacusine, tetrahydrobisbenzylisoquinoline alkaloids, antibacterial, COX-2, anti-inflammatory, molecular docking, molecular dynamics simulation

## Abstract

*Phaeanthus ophthalmicus* (Roxb. ex G.Don) J.Sinclair (previously known as *P. ebracteolatus* (Presl) Merr) is a Philippine medicinal plant occurring as evergreen shrub in the lowland forests of Luzon islands. It is used traditionally by Filipinos to treat bacterial conjunctivitis, ulcer and wound infections. Based on previous investigations where cyclooxygenase-2 (COX-2) functions as immune-linked factor in infectious sensitivities to bacterial pathogens by triggering pro-inflammatory immune-associated reactions, we investigated the antimicrobial and COX inhibitory activities of the extracts and tetrahydrobisbenzylisoquinoline alkaloids of *P. ophthalmicus* in vitro and in silico to validate its ethnomedicinal uses. Thus, the dichloromethane–methanol (DCM–MeOH) crude extract and alkaloid extracts exhibiting antibacterial activities against drug-resistant bacterial strains such as methicillin-resistance *Staphylococcus aureus* (MRSA), vancomycin-resistant *Enterococcus* (VRE), *Klebsiella pneumoniae* + CRE and *Pseudomonas aeruginosa* + MBL afforded (+)-tetrandrine (**1**) and (+)-limacusine (**2**) as the major biologically active tetrahydrobisbenzylisoquinoline alkaloidal constituents after purification. Both tetrahydrobisbenzylisoquinoline alkaloids **1** and **2** showed broad spectrum antibacterial activity with strongest inhibition against the Gram-negative bacteria MβL-*Pseudomonas aeruginosa Klebsiella pneumoniae* + CRE. Interestingly, the alkaloid limacusine (**2**) showed selective inhibition against ovine COX-2 in vitro. These results were ascertained by molecular docking and molecular dynamics simulation experiments where alkaloid **2** showed strong affinity in the catalytic sites of Gram-negative bacterial enzymes *P. aeruginosa* elastase and *K. pneumoniae* KPC-2 carbapenemase (enzymes involved in infectivity mechanisms), and of ovine COX-2. Overall, our study provides credence on the ethnomedicinal use of the Philippine medicinal plant *P. ophthalmicus* as traditional plant-based adjuvant to treat bacterial conjunctivitis and other related infections. The antibacterial activities and selective COX-2 inhibition observed for limacusine (**2**) point to its role as the biologically active constituent of *P. ophthalmicus.* A limited number of drugs with COX-2 inhibitory properties like celecoxib also confer antibacterial activity. Thus, tetrahydrobisbenzyl alkaloids, especially **2**, are promising pharmaceutical inspirations for developing treatments of bacterial/inflammation-related infections.

## 1. Introduction

The resurgence of infections caused by multi-drug resistant bacterial pathogens poses great threats globally and constitutes a major crisis plaguing societies in the past years [1]. Many pathogenic bacteria such as *Staphylococcus aureus* and *Pseudomonas aeruginosa* exhibit multi-drug resistance (MDR). Recently, a “pan-resistant” strain of the Gram-negative bacterium P. aeruginosa emerged and caused alarming consciousness of its infectivity [2,3,4]. Therefore, new drugs and treatment regimens are increasingly needed to catch up with the rise of bacterial resistance. While there are available antibiotics used to treat bacterial infections, the need for antimicrobials that regulate host immune response and diminish inflammation incidences are also increasing in demand. Recent efforts illustrate COX-2 inhibitors (i.e., celecoxib) confer increased bacterial sensitization and reversal of drug resistance [5,6]. This antibiotic drug discovery strategy plays a significant role in finding treatments for inflammatory-associated infections such as bacterial conjunctivitis and corneal ulcers. Thus, efforts to discover and develop drugs that exhibit both antibacterial and anti-cyclooxygenase activity are warranted. Plant-based natural products have been considered safe traditional remedies to treat bacterial infections [7]. For example, the extracts of the anti-inflammatory plant *Calendula officinalis* are applied topically to treat conjunctivitis [8] while extracts of *Jasminum* flowers possess antibacterial and antifungal properties relevant for treating inflamed eyes [9].

*Phaeanthus* Hook.f & Thomson is among the one hundred thirty-five genera of the family Annonaceae and comprised of thirty-six species; however, only four are currently accepted, while the rest are still unresolved [10]. In the Philippines, the species can be sometimes mistaken as *Uvaria* Roxb. ex G. Don or *Goniothalamus* (Blume) J.D. Hook. & Thomson due to the similar appearance of their fruits and flowers.

**Figure 1:**
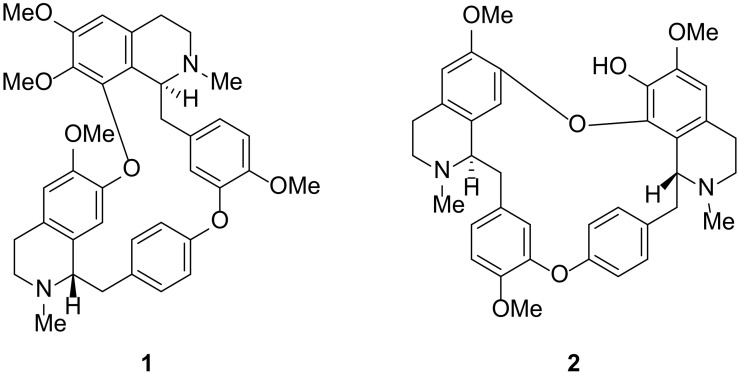
Tetrahydrobisbenzylisoquinoline alkaloids tetrandrine (**1**) and limacusine (**2**) from *Phaeanthus ophthalmicus*.

The Philippine medicinal plant *Phaeanthus ophthalmicus* (Roxb. ex G.Don) J.Sinclair (Annonaceae) locally known as “*kalimatas*” in Filipino (synonymous to *P. cumingii* Miq.; *P. ebracteolatus* (C.Presl) Merr.; *Uvaria ebracteata* C.Presl; *U. ebracteolata* C.Presl; *U. ophthalmica* Roxb. ex G.Don) is an evergreen shrub that grows in lowland forests of Luzon island, Philippines. It is characterized by inner petals that are longer than outer petals, numerous carpels and stamens, and monocarpous fruits. *P. ophthalmicus* is previously known as the endemic *P. ebracteolatus* (C.Presl) Merr. The leaves and bark of *P. ophthalmicus* are traditionally used in the Philippines to treat pink eyes or “sore eyes” by placing a drop of an aqueous extract into the sore eyes with inflamed conjunctiva [11]. It is also used traditionally to alleviate muscular spasms, hyperacidity/stomach ulcers and minor wounds [12]. The extracts and compounds of *P. ophthalmicus* exhibit photosensitizing effects useful in photodynamic therapies [13]. As part of our growing interest to validate the ethnomedicinal use of Philippine Annonaceae plants [14,15,16,17,18,19], we herein report the inhibitory activity of *P. ophthalmicus* extracts and tetrahydrobisbenzylisoquinoline alkaloids tetrandrine (**1**) and limacusine (**2**) (Figure 1) against drug resistant bacterial strains in addition to their cyclooxygenase (COX-1 and -2) inhibitory activities. To probe on the (selective) binding affinity and to depict binding mechanism of antimicrobial and COX-2 inhibitory activity, molecular docking studies and all-atom molecular dynamics simulation experiments were performed against bacterial enzymes and ovine COX isoenzymes.

## 2. Results and Discussion

### 2.1. Antibacterial Activity of Extracts and Alkaloids

In this study, antimicrobial activity was initially investigated on the *Phaeanthus ophthalmicus* extracts and alkaloids. Thus, the crude dichloromethane–methanol (DCM-MeOH) extract, crude alkaloid sub-extracts and tetrahydrobisbenzylisoquinoline alkaloids tetrandrine (**1**) and limacusine (**2**) were evaluated for antibacterial activity against four antibiotic-resistant commonly observed pathogens (Table 1). These multi-drug resistant (MDR) bacterial isolates were provided by the Makati Medical Center, Makati City, Philippines. Minimum Inhibitory Concentration (MIC) was determined by selecting the lowest concentration of test samples that completely inhibited the growth of the bacteria in microwell plates.

Test samples showed inhibitory activity against the test bacterial organisms with MIC values from 68.75 to 275 μg/mL (Table 1). The crude DCM-methanol extract (Po) exhibited MIC values from 137.5 to 275 μg/mL. Fractionation of the crude extract by gradient pH acid-base partitioning yielded the alkaloid-rich sub-extracts PoA, PoB and the non-alkaloid extract, PoE. Sub-extract PoB showed broad spectrum, and two-fold improvement of MIC (68.75 μg/mL) against Gram-negative MDR bacteria especially against *K. pneumoniae* and *P. aeruginosa* demonstrating selectivity on these test organisms.

To determine the biologically active components, the major tetrahydrobisbenzylisoquinoline alkaloids **1** and **2** were isolated, purified and identified. Identification of the alkaloids was facilitated by spectroscopic methods and by comparison with published spectroscopic data [20,21]. An MIC of 68.75 μg/mL was noted against *K. pneumoniae* + CRE and *P. aeruginosa* + MBL and, 137.5 μg/mL against MRSA and VRE for each alkaloid (Table 1). On the other hand, a higher MIC (137.5 μg/mL) was required to inhibit the Gram-positive MRSA and vancomycin resistant *Enterococcus.*

Minimum bactericidal concentrations (MBC) on each of the fractions and two isolated alkaloids were also obtained (Table 1). An MBC of 68.75 μg/mL was observed against gram-negative *K. pneumoniae* + CRE and *P. aeruginosa* + MBL. Higher MBC (137.5 μg/mL) was noted in both MRSA and VRE.

Results of the antibacterial assays suggest the potential of tetrandrine (**1**) and limacusine (**2**) from *P. ophthalmicus* against drug-resistant Gram-negative bacterial strains. Antibiotic-resistant Gram-negative bacteria possess different antibiotic resistance mechanisms including acquisition of drug-destroying enzymes, and mutations in drug targets like topoisomerases and outer membrane porins leading to lesser antibiotic efficacy and uptake. These mechanisms are transmitted mostly to other bacteria through genetic mobile elements [22,23]. Since lesser number of new antibiotics targeting Gram-negative bacteria are being developed coupled with the rise of number of infections associated with multidrug-resistant Gram-negative bacteria, development of natural products which may selectively inhibit or kill these bacteria is warranted to enter clinical trials.

Alkaloids have a proven reputation in the development of antibacterial and other chemotherapeutic compounds. In the case of tetrahydrobisbenzylisoquinoline dimers like tetrandrine (**1**) and limacusine (**2**), the presence of rigid aromatic rings and centrally locked nitrogen atoms are fundamental for improved antibacterial activity [24]. The occurrence of basic nitrogen atoms and their capability to accept protons, and amine donating hydrogen atoms which is accompanied by proton-acceptor and -donor functional provides unique bioactivity to several types of alkaloids [25]. Mechanism of action typically anchors on synergism through perturbation of bacterial efflux pumps. Relevant to this study, anti-MRSA synergistic effects between dimeric tetrahydrobisbenzylisoquinoline alkaloids such as tetrandrine (**1**) and commonly used antibacterial drugs have been observed to enhance in vitro inhibitory efficacy for example of the antibiotic drug, cefazolin [26]. Isoquinoline alkaloids are known to exert bactericidal effects by inhibiting bacterial nucleic acid synthesis and cell division [27,28,29,30]. Antibacterial mechanism of action of alkaloids differs for each type. Interestingly, tetrandrine (**1**) and limacusine (**2**) showed selective antibacterial activity against Gram-negative bacteria. Due to the presence of outer membrane in Gram-negative bacteria which provides additional protection against entry of antibacterial agents, mechanisms of antibiotic alkaloids include (1) disruption of outer membrane, (2) diffusion along outer membrane lipid components, and (3) movement through the outer membrane via hydrophilic porins [23].

The drug development of efficient and cost-effective treatment modalities for bacterial conjunctivitis is an interesting field in ophthalmic disease drug discovery, where current treatment armamentariums are challenged by resurgence of pathogens and rapid emergence of drug resistant strains. Therefore, the development of safe and effective natural products that can enter clinical trials is warranted to curb the emergence and reemergence of these pathogens. While antibacterial activities of *P. ophthalmicus* constituents, such as *O*-methyldauricine, corydaldine, limacine, and oxostephanine, have been reported [31], our study reports for the first time the identification and anti-MDR bacterial properties of tetrandine (**1**) and limacusine (**2**). This result could be used in conjunction with other studies in which various natural products have demonstrated anti-bacterial activities against drug resistant strains.

### 2.2. Cyclooxygenase (COX) -1 and -2 Inhibitory Activity of Alkaloids

The manner by which COX-1 and COX-2 are expressed in tissues sheds light towards greater understanding of their respective functions. Low level constitutive and stable expression of COX-1 in most tissues allow constant production of prostaglandin necessary to maintain essential physiological functions such as platelet aggregation, renal water balance and gastric mucosal protection. On the other hand, COX-2 is mostly silent, but its expression can be triggered in response to pathogens [32]. Guided by the fact that bacterial conjunctivitis is a chronic inflammatory disease that can be caused by bacterial pathogens, it is important to evaluate the inhibitory potential of the alkaloids on both COX isoforms, with better selectivity against COX-2. Thus, the anti-inflammatory properties of the isolated alkaloids **1** and **2** were investigated by examining their peroxidase activity against the ovine cyclooxygenase isozymes, COX-1 and -2. Limacusine (**2**) showed selectivity against the COX-2 isoform compared to tetrandrine (**1**) (Table 2). Both compounds exhibited modest COX-1 and -2 activities compared to the positive drug control celecoxib.

### 2.3. Molecular Docking of Alkaloids ***1*** and ***2*** against Disease Targets

Results of antibacterial assay showed potential of tetrandrine (**1**) and limacusine (**2**) against Gram-negative bacteria. Thus, both compounds were docked against selected target enzymes present in Gram-negative *P. aeruginosa* and *K. pneumoniae* specifically *P. aeruginosa* FabA, LasR and elastase, and *K. pneumoniae* KPC-2 carbapenemase and CTX-M-15 protein (Table 2). Both tetrandrine (**1**) and limacusine (**2**) showed highest binding affinities against *P. aeruginosa* elastase (or LasB) with binding energies of −8.1 kcal/mol and −7.2 kcal/mol, respectively. Both alkaloids also exhibited highest binding propensities against *K. pneumoniae* KPC-2 carbapenemase with binding energy of −7.6 kcal/mol for tetrandrine while −9.0 kcal/mol for limacusine.

**Figure 2:**
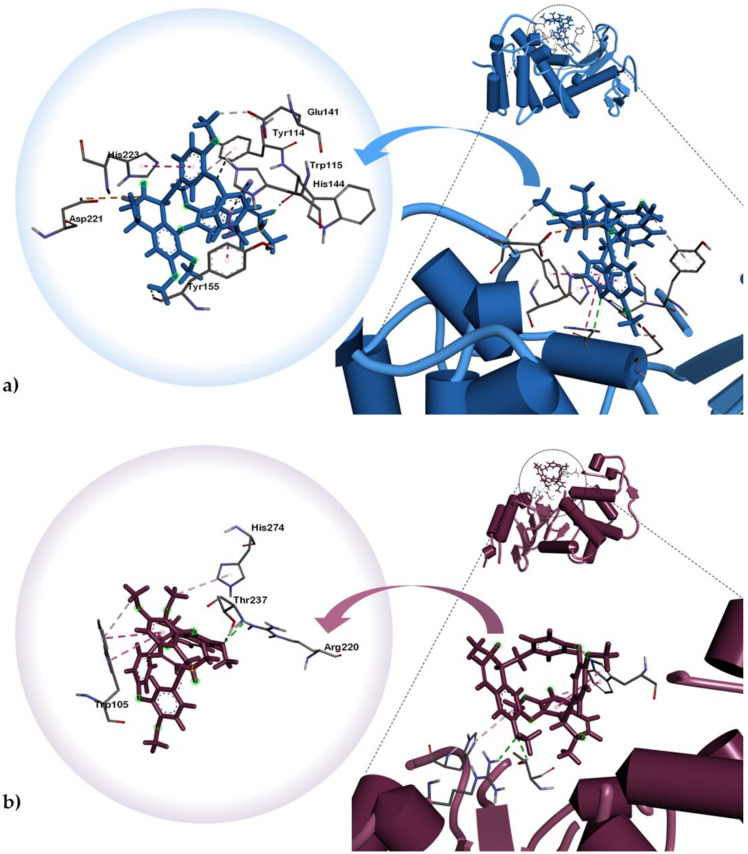
Dock poses of alkaloid **1** against (**a**) *P. aeruginosa* elastase (PDB ID: 1U4G) and (**b**) *K. pneumoniae* KPC-2 carbapenemase (PDB ID: 2OV5).

**Figure 3:**
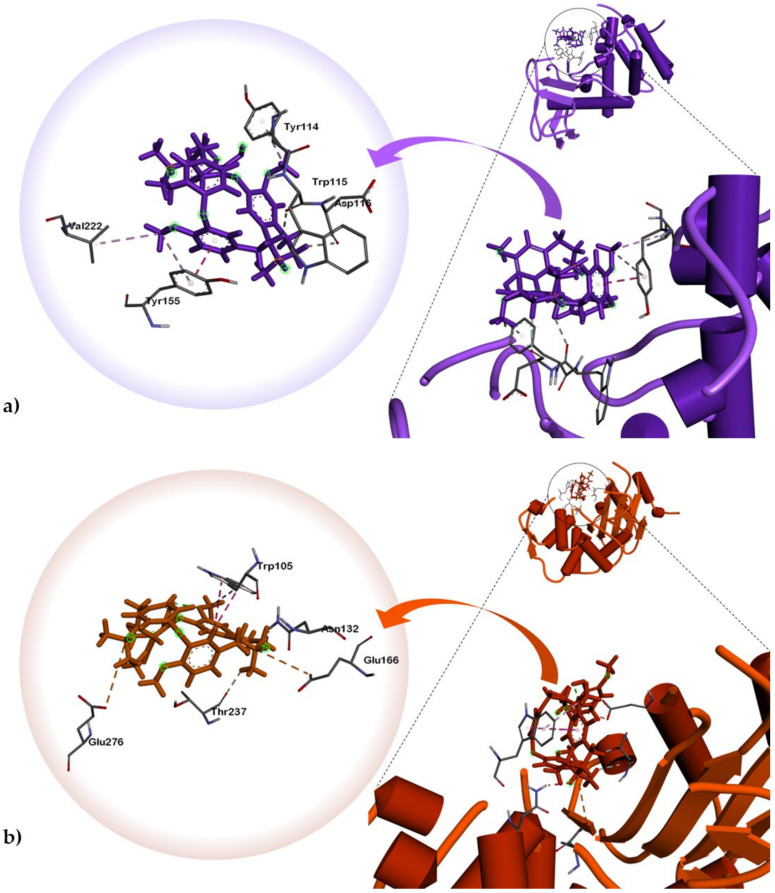
Dock poses of alkaloid **2** against (**a**) *P. aeruginosa* elastase (or LasB) (PDB ID: 1U4G), (**b**) *K. pneumoniae* KPC-2 carbapenemase (PDB ID: 2OV5).

Tetrandrine (**1**) interacted with *P. aeruginosa* elastase via attractive charge between Asp221 and isoquinoline B nitrogen atom (Figure 2). This interaction is stabilized in the ligand-binding cavity through *pi-pi* (T-shaped) and *pi-pi* alkyl interactions with His223 and Tyr114. Most notably, hydrogen bonds were observed between the nitrogen substituent of isoquinoline A and TrpA (Table 3). One of the phenoxide moieties exhibited hydrogen bonding with His144 and Van der Waals (VdW) interaction with Tyr155. Alkaloid **1** is stabilized in *K. pneumoniae* KPC-2 carbapenemase binding pocket via *pi-pi* (T-shaped) interaction between Trp105 of the phenoxide moiety of isoquinoline B and *pi*-alkyl interaction between His274 and phenoxy ether of isoquinoline B. Thr237 and Arg220 also exhibited hydrogen bonding with the methoxy groups of the isoquinoline A phenoxide moiety. Meanwhile, docking simulations of limacusine (**2**) against *P. aeruginosa* LasB demonstrated VdW interaction with Trp115 and Asp116 of the isoquinoline A nitrogen and alkyl substituents, respectively (Table 3, Figure 3). A *pi-pi* stacking interaction between Tyr155 and methoxy substituent of one of the single aromatic rings was also observed. For *K. pneumoniae* KPC-2 carbapenemase, alkaloid **2** exhibited two attractive charges with Glu166 and Glu276 with the nitrogen atoms of isoquinoline rings A and B, respectively. An intramolecular VdW was also observed between Thr237 and the alkyl substituent of isoquinoline A. A *pi-pi* stacked interaction between Trp105 and phenoxide moiety of isoquinoline A was also noted.

The high binding affinities and binding interactions of tetrandrine (**1**) and limacusine (**2**) observed with *P. aeruginosa* elastase and *K. pneumoniae* KPC-2 carbapenemase enzymes may support mechanisms underlying their Gram-negative bacterial inhibitory activity. LasB is among the virulence factors of *P. aeruginosa* and is responsible for corneal tissue degradation as well as destruction of collagen and elastin [33]. Given that decreasing virulence factor activity results in lesser antibiotic resistance in *P. aeruginosa* [34,35], elastase is an important antibacterial target. On the other hand, *K. pneumoniae* KPC-2 carbapenemase is involved in multiple antibiotic resistance mechanisms of *K. pneumoniae* along with its variant KPC-3 [36,37]. Carbapenemases bind with and hydrolyze carbapenems, a known group of antibiotics against *Klebsiella* infections [38]. Thus, development of inhibitors based on carbapenem-hydrolyzing enzymes and tetrahydrobisbenzylisoquinoline alkaloids from *P. ophthalmicus*, especially limacusine (**2**), with KPC-2 inhibitory activity in silico and anti-Gram-negative bacterial activity in vitro is a promising approach.

**Figure 4:**
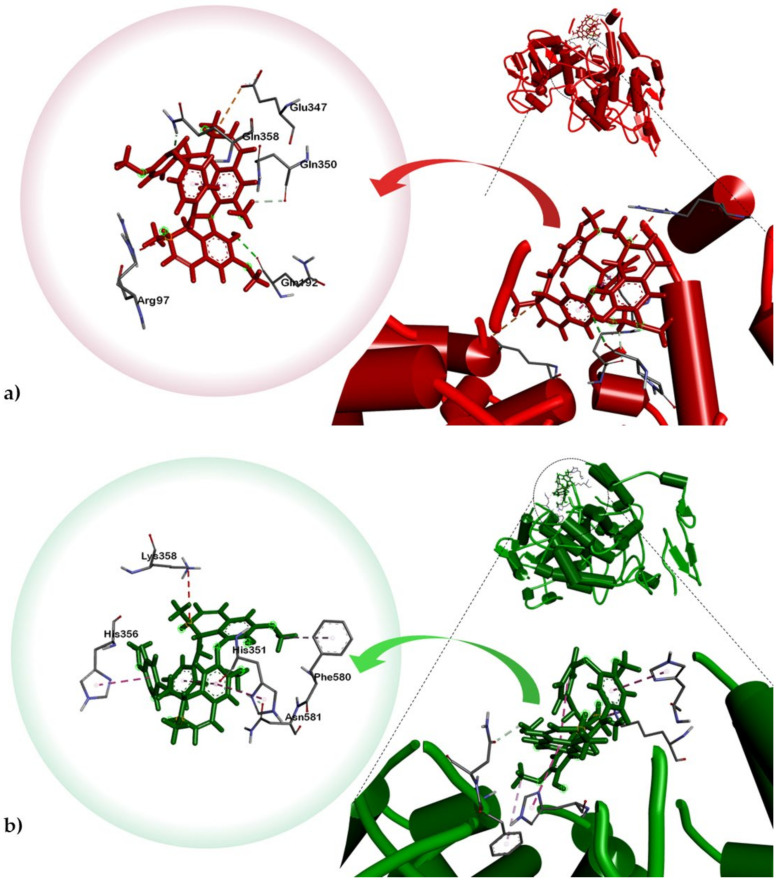
Dock poses of alkaloid **2** against ovine (**a**) COX-1 (PDB ID: 3KK6) and (**b**) COX-2 (PDB ID: 4M11).

Both compounds were also docked onto COX-1 and COX-2. Thus, results of our COX assay were further supported by our molecular docking analysis which showed lower binding energy for limacusine (**2**) towards COX-2 compared to tetrandrine (**1**) (Table 2). The cyclooxygenase active site is created by a long hydrophobic channel, which the substrate-binding pocket is predominantly lined by hydrophobic residues. This is the site for most non-steroidal anti-inflammatory drug binding [39,40]. However, the attachment of alkaloid **2** is not on the hydrophobic channel but rather on a larger neighboring hydrophilic side pocket (Figure 4). Compound **2** is stabilized to the said hydrophilic pocket mostly via *pi*-*pi* (T-shaped) stacking interactions with His351 and His356 against the isoquinoline A and phenoxide moieties, respectively (Table 2). A *pi*-alkyl interplay can also be observed between one of the methoxy substituents of isoquinoline B and Phe580 as well as an intramolecular C-H bonding between an OH group and Asn581. The presence of a hydroxyl substituent in **2** and the fact that it is more polar than **1** (based on the topological polar surface area, TPSA, estimated using Osiris Property Explorer) (Supplementary Table S2) could be the primary reason for the stronger binding affinity of limacusine (**2**) with the hydrophilic binding pocket of COX-2. Both compounds were also docked against COX-1. Compound **1** exhibited a weaker affinity towards the said enzyme (BE = −7.4 kcal/mol) compared to compound 2 (BE = −8.8 kcal/mol). Based on these results, tetrandrine (**1**) consistently showed weaker binding to both COX-1 and COX-2, consistent with the results of the in vitro data. In the same regard, the results of the in vitro anti-COX assay for limacusine (**2**) correlates with in silico analysis showing selectivity towards COX-2 over COX-1.

### 2.4. Molecular Dynamics Simulation on Limacusine-COX-2 Docked Complex

**Figure 5:**
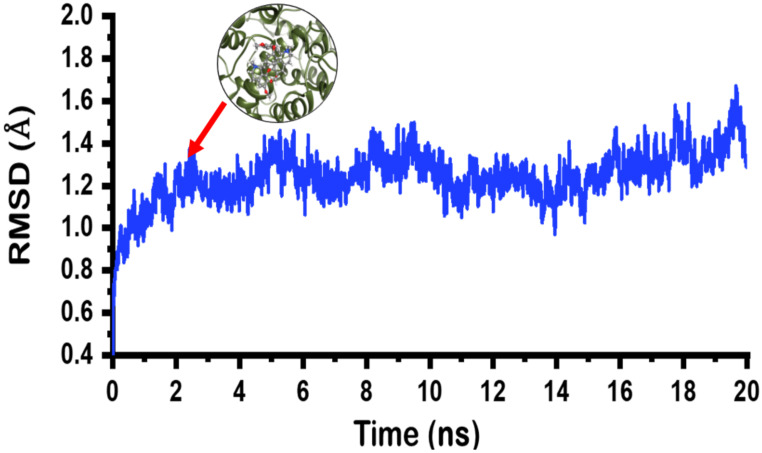
RMSD of the docked poses of limacusine (**2**). The *x*-axis is showing time in nanoseconds while the *y*-axis is showing RMSD in angstrom (Å).

The docked limacusine-COX-2 complex was subjected to molecular dynamics simulations to determine its stability. Results from molecular dynamics simulation were retrieved and analyzed. For stability, the root mean square deviation or RMSD was calculated while residual flexibility root mean square fluctuation (RMSF) was accessed. The RMSD results (Figure 5) revealed that the limacusine-COX-2 docked complex attained stability soon after reaching 2 ns. The stability was attained at 1 Å. The average RMSD for the complex was reported to be 1 Å. Hence, these results confirm that the binding of alkaloid **2** inside the binding remained stable during the simulation time.

**Figure 6:**
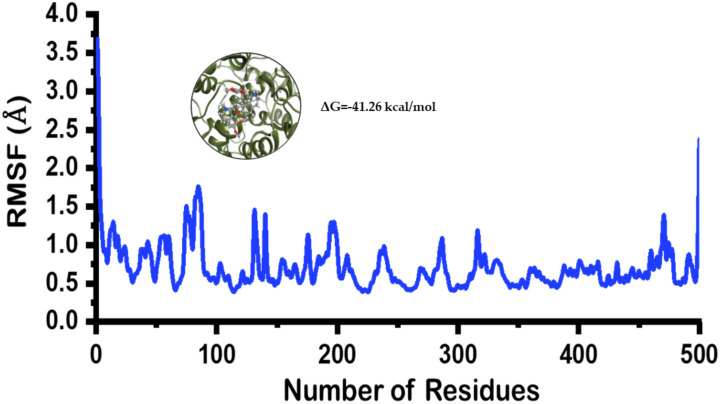
RMSF of the docked poses of limacusine. The *x*-axis is showing the total number of residues while the *y*-axis is showing RMSF in angstrom (Å).

To understand the residual flexibility pattern, it can be seen that regions 80–100, 140–150 and 180–220 fluctuated a little higher than the others. While the flexibility of other residues remained lower (Figure 6). Furthermore, the binding affinity analysis revealed stronger binding for alkaloid **2** in the active site. The total binding energy (ΔG) for this complex was reported to be −41.26 kcal/mol. The electrostatic energy was reported −16.42 kcal/mol while the vdW was reported to be −36.24 kcal/mol. Hence, these results suggest alkaloid **2** efficiently binds and inhibit the COX-2 active site functionalities.

A prediction of the toxicities, such as mutagenicity, tumorigenicity, irritant effect and reproductive toxicity, of compounds **1** and **2** was also performed using OSIRIS Property Explorer [41]. As presented in Supplementary Table S2, both compounds were predicted to show no to low toxicity risks.

Taken together with the results of antibacterial and COX inhibitory assays, limacusine (**2**) was shown to be the active alkaloidal component implicated in the traditional use of *P. ophthalmicus* as cure for eye bacterial conjunctivitis. Our study may also provide a basis on the ethnomedicinal use of *P. ophthalmicus* in treating ulcer, another inflammatory illness. Alkaloid **2** is a promising 2-in-1 natural product platform with antibacterial and anti-inflammatory properties.

## 3. Materials and Methods

### 3.1. General

Optical rotation measurements were performed using a Krüss Optronic GmbH spectropolarimeter. Ultraviolet (UV) spectroscopic analysis was done using Perkin Elmer lambda 25 spectrophotometer. Infrared (IR) spectral data were obtained using Shimadzu IR Prestige-21 spectrophotometer coupled with Diffused Reflectance Spectroscopy (DRS) in KBr. Nuclear magnetic resonance (NMR) spectroscopic data were obtained using Agilent DD2 MR Varian (500 MHz for ^1^H NMR and 125 MHz for ^13^C NMR). Samples were dissolved in deuterated chloroform (CDCl_3_) with tetramethylsilane (TMS) as internal standard. Column chromatography was performed on silica gel Merck 60 F_254_ [(0.2–0.5 mm) and (0.2–0.063 mm)] 70–230 and 230–400 mesh (Darmstadt, Germany). Pre-coated silica gel 60 F_254_ thin-layer chromatography (TLC) plates (Merck, Darmstadt, Germany) were used for monitoring purification and compounds were visualized under UV light (254 and 365 nm) and stained with Dragendorff’s reagent.

### 3.2. Plant Material

The mature leaves of *Phaeanthus ophthalmicus* were collected from Tibiao, Antique, Philippines (N 11′18; E 122′04; altitude: 210 m asl) during summer season of May, 2015. A voucher specimen (UST-15-623B) was deposited at the University of Santo Tomas Herbarium (USTH), Research Center for the Natural and Applied Sciences (RCNAS), University of Santo Tomas, Manila. Taxonomic identification was conducted using both morphological and molecular phylogenetic methods. Fresh leaves were air-dried at room temperature, and ground using Wiley Mill with fine mesh. The powdered plant material (4 kg) was extracted with 1:1 dichloromethane–methanol (DCM-methanol) (40 L) and concentrated under reduced pressure to yield the crude extract (400 g) [42].

### 3.3. Extraction, Isolation and Structure Elucidation of Compounds ***1*** and ***2***

A portion of the crude extract (Po, 300 g) was subjected to gradient pH acid (1M H_2_SO_4_—base (sodium carbonate) extraction [43] to obtain two crude alkaloid extracts PoA (85 g, extracted at pH 5) and PoB (98 g, extracted at pH 9) along with the non-alkaloid crude extract (62 g, PoE) [44]. The PoB extract was fractionated using silica gel vacuum liquid chromatography with increasing gradients (20%) of ethyl acetate in methanol to give three fractions, PoB.1 to PoB.3. Fraction PoB.1 (1.72 g) was subjected to silica gel column chromatography (3x) eluted with 1:1 ethyl acetate-methanol to yield compound **1** as a white amorphous powder (319 mg).

The second fraction PoB.2 (47.6 g) was subjected to silica gel vacuum liquid chromatography. Elution was carried-out with 9:1 followed by 1:1 ethyl acetate-methanol to afford seven fractions. The third sub-fraction, PoB2.3 (14 g) was again column chromatographed in silica gel with 1:1 ethyl acetate-methanol to afford compound **2** (49 mg).

(+)*-Tetrandrine* (**1**): Needle-like crystals (319.0 mg); [α]_24_ D +140° (*c* 0.0004, MeOH); UV (MeOH) λmax (log ε) 235 (3.81), 281 (3.45) nm; IR (KBr) *ν*_max_ 2935, 2837, 1577, 1507, 1457, 1417, 1268, 1125, 1025 cm^−1^.

(+)*-Limacusine* (**2**): Needle-like crystals (49.0 mg); [α]_24_ D +90° (*c* 0.0008, MeOH); UV (MeOH) λmax (log ε) 204 (1.81), 280 (0.77) nm; IR (KBr) *ν*_max_ 3323 (br), 2940, 2832, 2368, 1583, 1509, 1464, 1445, 1273, 1126, 1024 cm^−1^. The ^1^H NMR and ^13^C NMR of compounds **1** and **2** are in good agreement with the literature [20,21].

### 3.4. Biological Assays

#### 3.4.1. Test Organisms and Growth Conditions

The test organisms for in vitro antibacterial screening included methicillin-resistant *Staphylococcus aureus*, CRE *Klebsiella pneumoniae*, vancomycin resistant *Enterococcus* and MβL-*Pseudomonas aeruginosa* (Table S1). MDR bacterial isolates were obtained from the Makati Medical Center (Makati City, Philippines). Colorimetric determination using the Vitek^®^MS gram-positive (GP) identification card (bioMérieux, Marcy l’Etoile, France) was used to identify all the isolates. The antibiotic resistance patterns of the isolates were detected using Vitek^®^MS AST (bioMérieux, Marcy l’Etoile, France) following MIC interpretive standards of the Clinical Laboratory Standard Institute M100-S25 [45].

#### 3.4.2. MIC Determination

The minimum inhibitory concentrations (MICs) of the Po crude extracts and compounds **1** and **2** were determined in sterile 96-well microplates using the broth microdilution folllwing the method of the Clinical Laboratory Standard Institute, M07-A8 [46]. Each test was done in triplicate. Test samples were serially diluted to produce final concentrations of 2.15 μg/mL to 550 μg/mL. Cation-adjusted Mueller-Hinton broth (Becton Dickinson and Company, Holdrege, Nebraska, United States) was used as diluent. The set-up included bacterial growth controls in wells containing 10 µL of the test inoculum and negative controls without bacterial inoculum. Reference drug controls were included in the set-up [47].

The inoculum was prepared by direct saline suspension of isolated bacterial colonies selected from an 18 to 24 h 5% sheep BAP culture. Suspension was adjusted to achieve a turbidity equivalent to 0.5 McFarland turbidity standard, which approximated 1.5 × 10^8^ cells/mL. Within 15 min after standardization, 10 µL of the adjusted inoculum was added to each well containing 100 µL test sample in the dilution series, and mixed. The sealed microdilution trays were incubated at 35 ± 2 °C for 16 to 20 h in an ambient air incubator.

MICs were determined by selecting the lowest concentration of Po extracts or compounds **1** and **2** that completely inhibited the growth of the organism in the well as detected by the unaided eye. To determine the growth endpoints, the amount of growth in the wells containing the Po extracts or compounds **1** and **2** were compared with the amount of growth in the growth-control well (no test samples) used in each set of tests. For a test to be considered valid, acceptable growth (≥2 mm button or definite turbidity) must occur in the growth-control well.

#### 3.4.3. MBC Determination

MBC was determined following the methods described by Irobi and Daramola [48] with slight modifications. Wells with no visible growth in MIC assays were sub-cultured using a 10 µL-inoculating loop onto a 5% sheep BAP at 35 ± 2 °C for 16 to 20 h incubation. MBC is defined as the lowest concentration of the test sample that did not permit any growth.

#### 3.4.4. Anti-Cyclooxygenase (COX) -1 and -2 Assays

The inhibitory assay against COX-1 and COX-2 isozymes was carried out using a Cayman anti-COX inhibitory compound screening kit. The assay measures peroxidase activity of cyclooxygenases by observing colorimetrically the oxidation of N,N,N′,N′-tetramethyl-*p*-phenylenediamine (TMPD) at 590 nm. The protocol indicated in the manufacturer’s manual was followed, and Po extracts and compounds **1** and **2** were dissolved in DMSO. Inhibitory activity was reported as IC_50_ in µM units.

### 3.5. Computational Methods

#### 3.5.1. Molecular Docking

Compounds **1** and **2** were subjected to molecular docking simulations against different disease targets to assess their binding characteristics. For *P. aeruginosa*, FabA (PDB ID: 4B8U; chain B), LasR (PDB ID: 3JPU; chain B), and elastase (or LasB) (PDB ID: 1U4G) were selected as target enzymes. Meanwhile, the following proteins were used for *K. pneumoniae* infection: KPC-2 carbapenemase (PDB ID: 2OV5) and CTX-M-15 enzyme (PDB ID: 4S2I; chain A). These enzymes are commonly targeted in silico for drug development due to their involvement in infectivity and/or multi-drug resistance mechanisms of the bacteria [49,50,51,52,53,54,55].

Both compounds were also docked against cyclooxygenases-1 (PDB ID: 3KK6) and -2 (PDB ID: 4M11). All enzymes were fetched from the protein data bank as co-crystallized structures. UCSF Chimera (version 1.14.1) (University of California-San Francisco, CA, United States) was used to facilitate the removal of bound residues and minimization of structures. Dock-prepping of ligand and protein structures was done using Antechamber and molecular docking was performed using the BFGS algorithm of AutoDock Vina (version 1.1.2) (Scripps Research, La Jolla, California, United States) [56,57]. Validation of the docking protocol was done via redocking experiment of the co-crystallized ligands. The conformational protein–ligand structure was visualized and analyzed using Biovia Discovery Studios (version 4.1) (Vélizy-Villacoublay, France). ORISIS Property explorer program 2017 (Thomas Sander, Idorsia Pharmaceuticals Ltd., Allschwil, Switzerland) was employed for in silico toxicity and related physicochemical property prediction [41,58].

#### 3.5.2. All Atoms Molecular Dynamics Simulation

To access the dynamics stability and behaviour of the more active compound **2** against COX-2, we subjected the docked complex to molecular dynamics simulations. Amber20 (University of California-San Francisco, California, United States) was used with FF14SB force field [59,60]. For the small molecule topology an integrated module antechamber was used [61]. For ligand, GAFF2 force field was used [62]. The system was neutralized by the addition of ions. To solvate the protein complex TIP3P waterbox module with 10.0 Å distance was used. Two steps gentle energy minimization was performed. In the first minimization 6000 steps while in the second minimization 4000 steps were completed. After minimization, these complexes were heated up to 300 K for 0.2 ns, and then we equilibrated the system with weak restraint and without restraint for 2 ns at 300 K, respectively. The temperature was controlled with Langevin thermostat [63], and the simulation was run for 20 ns. Long-range electrostatic interactions were detected with the particle mesh Ewald method using a cutoff distance of 10.0 Å. SHAKE method [64] was applied for covalent bond treatment. MD simulation production step was performed on GPU supported pmemd code for each system, and the trajectories were analyzed on Cpptraj and Ptraj package in Amber 20 [65]. To further understand the real time binding affinity of the compound **2** to the two enzymes, we calculated the binding free energy using MMPBSA.py script. This method is widely implemented in such studies to understand the binding of drug, protein, DNA or RNA [66,67,68,69,70,71]. The total energy was calculated for the ligand.

## 4. Conclusions

The present study demonstrated the antibacterial and anti-inflammatory potentials of tetrahydrobisbenzylisoquinoline alkaloids especially limacusine (**2**) as well as the crude extracts and alkaloid sub-extracts of the Philippine medicinal plant *Phaeanthus ophthalmicus*. The results of our work are consistent with other studies implicating the relevance of COX-2 inhibition with antibacterial activity. It also corroborates with previous investigations where modulatory roles of the COX-2 biochemical cascade during bacterial infection in *Pseudomonas aeruginosa* are explained [72]. The selective inhibition of COX-2 by limacusine (**2**) correlates to its observed biological activity against bacterial pathogens causing inflammatory conjunctivitis. Future studies may be done to confirm whether COX-2 inhibition will cause complete bacterial inhibition. Additionally, our results suggest that inhibiting COX-2 is not only crucial to control bacterial conjunctivitis but also other bacterial infectious diseases. The antibacterial activities along with a report on the selective COX-2 inhibitory activity of limacusine (**2**) support and validate the traditional use of *P. ophthalmicus* in treating conjunctivitis and other related bacterial infections.

## Figures and Tables

**Table 1:** Minimum inhibitory concentrations (MIC) & minimum bactericidal concentrations (MBC) against multidrug-resistant bacterial isolates, and cyclooxygenase (COX) inhibitory activity of *P. ophthalmicus* extracts and alkaloids **1** and **2**.

Tested Samples	MIC and MBC (μg/mL)								IC_50_ (μM) vs. COX	
	MRSA		VRE		*K. pneumoniae*, + CRE		*P. aeruginosa*, + MBL		COX-1	COX-2
	MIC	MBC	MIC	MBC	MIC	MBC	MIC	MBC		
Po	275	275	137.5	275	137.5	137.5	137.5	137.5	-	-
PoA	137.5	275	137.5	275	137.5	137.5	68.75	137.5	-	-
PoB	137.5	137.5	137.5	137.5	68.75	137.5	68.75	68.75	-	-
PoE	137.5	137.5	137.5	137.5	137.5	137.5	68.75	68.75	-	-
**1**	137.5	137.5	137.5	137.5	68.75	68.75	68.75	68.75	>100	>100
**2**	137.5	137.5	137.5	137.5	68.75	68.75	68.75	68.75	>100	68.8
Penicillin	14	14	12	12	-	-	-	-	-	-
Meropenem	-	-	-	-	43.75	43.5	12	12.5	-	-
Celecoxib	-	-	-	-	-	-	-	-	6.9	0.9

(-) = not determined.

**Table 2:** Binding affinities of *P. ophthalmicus* alkaloids against target proteins.

Test Compounds	Binding Energy (kcal/mol)						
	*P. aeruginosa* Enzymes			*K. pneumoniae* Enzymes		Ovine Cyclooxygenase	
	FabA	LasR	Elastase (LasB)	KPC-2 carbapenemase	CTX-M-15	COX-1	COX-2
**1**	−6.3	−5.8 *	−8.1	−7.6	−6.5 *	−7.4	−7.8
**2**	−6.9	−6.5 *	−7.2	−9.0	−6.6 *	−8.8	−9.0
Meropenem	−6.7	−6.7	−5.3	−5.9	−6.6	-	-
Celecoxib	-	-	-	-	-	−9.9	−11.9

* Compound binds in the peripheral portion of the binding site.

**Table 3:** Interacting residues between alkaloids **1** and **2** and top target proteins.

Docked Ligands	Ovine COX-2		*P. aeruginosa* Elastase (LasB)		*K. pneumoniae* KPC-2 Carbapenemase	
	Hydrogen Bond	Other Interactions	Hydrogen Bond	Other Interactions	Hydrogen Bond	Other Interactions
Tetrandrine (**1**)	Asn104	Asn581, Glu346, Asp347, Lys97 (hydrophobic), Lys358 (*pi*-cation)	His144, Trp115	Glu141, Tyr155 (hydrophobic), Asp221 (attractive charge), His223 (*pi-pi* stacked), Tyr114 (*pi*-alkyl)	Thr237, Arg220	Trp105 (*pi-pi* T-shaped), His274 (*pi*-alkyl)
Limacusine (**2**)	None	His351, His356 (*pi-pi* T-shaped)	None	Val222, Tyr114 (*pi*-alkyl), Tyr155 (*pi-pi* stacked), Trp115, Asp116 (hydrophobic)	None	Glu276, Glu166 (attractive charge), Thr237 (hydrophobic), Trp105 (*pi-pi* stacked)

## Data Availability

Data sharing not applicable.

## Supplementary Materials

The following are available online at https://www.mdpi.com/2223-7747/10/3/462/s1, Table S1: Panel of multidrug-resistant (MDR) test bacteria, Table S2: Predicted toxicity and physical parameters of compounds **1** and **2**.
