# Supplementary material for: Antibacterial and COX-2 Inhibitory Tetrahydrobisbenzylisoquinoline Alkaloids from the Philippine Medicinal Plant Phaeanthus ophthalmicus
Source: Plants (Basel). 2021 Mar 1;10(3):462. doi: 10.3390/plants10030462 (PMC7999448; doi:10.3390/plants10030462)
Supplement: Supplementary file 1 [file plants-10-00462-s001.pdf]

Antibacterial and COX-2 inhibitory tetrahydrobisbenzylisoquinoline alkaloids from the Philippine medicinal plant *Phaeanthus ophthalmicus*

Hilbert D. Magpantay [1,†], Ivane N. Malaluan [2,3,†], Joe Anthony H. Manzano [2,4], Mark Tristan Quimque [2,5], Kirstin Rhys Pueblos [2,5], Natalija Moor [6], Simon Budde [6], Porferio S. Bangcaya [7], Demi Lim-Valle [8], Hans-Martin Dahse [9], Grecebio Jonathan D. Alejandro [10], Allan Patrick G. Macabeo [2,*]

[1] Chemistry Department, De La Salle University, 2401 Taft Avenue, 0922 Manila, Philippines

[2] Laboratory for Organic Reactivity, Discovery and Synthesis (LORDS), Research Center for the Natural and Applied Sciences, University of Santo Tomas, España Blvd., Manila 1015, Philippines

[3] Chemistry Department, College of Science, Bicol University, Rizal St., 4500 Legazpi City, Philippines

[4] Department of Biological Sciences, College of Science, University of Santo Tomas, España Blvd., Manila 1015, Philippines

[5] Chemistry Department, College of Science, MSU-Iligan State University, Iligan City, Philippines

[6] Institut für Organische Chemie, Universität Regensburg, D-93053 Regensburg, Germany

[7] Biological Science Department, College of Teacher Education – University of Antique, Tario-Lim Memorial Campus, Tibiao, 5707 Antique, Philippines

[8] Clinical Microbiology Laboratory, Department of Pathology and Laboratories, Makati Medical Center, Amorsolo St., Legaspi Village, 1229 Makati City, Philippines

[9] Leibniz-Institute for Natural Product Research and Infection Biology, Hans-Knöll-Institute (HKI), D-07745 Jena, Germany

[10] Plant Sciences Laboratory, Research Center for the Natural and Applied Sciences, University of Santo Tomas, España Blvd., Manila 1015, Philippines

* Correspondence: agmacabeo@ust.edu.ph; Tel.: (+632-4061611 ext. 4057 Fax.: +632-7314031)

† Both authors equally contributed to this study

Table S1. Panel of multidrug-resistant (MDR) test bacteria.

Characteristics	Species	Antibiotic resistance pattern
Gram-positive coccus, MDR, #1	MRSA	SXT, FOX, OX, P
Enterobacteriaceae, encapsulated, MDR	CRE, *Klebsiella pneumoniae*	AM, FEP, CTZ, CRO, IPM, MEM
Vancomycin-resistant *Enterococcus*	VRE	P, VA
Non-Enterobacteriaceae, MDR	MβL-*Pseudomonas aeruginosa*	FEP, CTZ, IPM, MEM

FOX- cefoxitin, OX- oxacillin, P – penicillin, SXT- Trimethoprim-sulfamethoxazole. AM- ampicillin, FEP- cefepime, CTX- cefotaxime, CTZ- ceftazidime, CRO- ceftriaxone, IPM- imipenem, MEM- meropenem, P – penicillin, VA- vancomycin.

Table S2. Predicted toxicity and physical parameters of compounds **1** and **2**.

Compound	Toxicity Parameters				Physical Parameters	
	Mutagenicity	Tumorigenicity	Irritant Effect	Reproductive Toxicity	clogP	TPSA
Tetrandrine (**1**)	none	none	none	none	6.50	61.86
Limacusine (**2**)	none	none	none	none	6.23	72.86
